# Arginine and Arginases Modulate Metabolism, Tumor Microenvironment and Prostate Cancer Progression

**DOI:** 10.3390/nu13124503

**Published:** 2021-12-16

**Authors:** Andreia Matos, Marcos Carvalho, Manuel Bicho, Ricardo Ribeiro

**Affiliations:** 1 Laboratory of Genetics and Instituto de Saúde Ambiental, Faculdade de Medicina, Universidade de Lisboa, 1649-028 Lisboa, Portugal; andreiamatos@medicina.ulisboa.pt (A.M.); marcoscarvalho511@gmail.com (M.C.); manuelbicho@medicina.ulisboa.pt (M.B.); 2 Instituto de Investigação Científica Bento da Rocha Cabral, 1250-047 Lisboa, Portugal; 3 Tumour & Microenvironment Interactions Group, INEB—Institute of Biomedical Engineering, i3S-Instituto de Investigação e Inovação em Saúde, Universidade do Porto, Rua Alfredo Allen 208, 4200-153 Porto, Portugal; 4 ICBAS-Institute of Biomedical Sciences Abel Salazar, University of Porto, R. Jorge de Viterbo Ferreira 228, 4050-313 Porto, Portugal; 5 Department of Pathology, Centro Hospitalar Universitário do Porto, 4099-001 Porto, Portugal; 6 S. Martinho Hospital, 4440-004 Valongo, Portugal

**Keywords:** arginine, arginase, metabolism, nitric oxide, prostate cancer, tumor microenvironment

## Abstract

Arginine availability and activation of arginine-related pathways at cancer sites have profound effects on the tumor microenvironment, far beyond their well-known role in the hepatic urea cycle. Arginine metabolism impacts not only malignant cells but also the surrounding immune cells behavior, modulating growth, survival, and immunosurveillance mechanisms, either through an arginase-mediated effect on polyamines and proline synthesis, or by the arginine/nitric oxide pathway in tumor cells, antitumor T-cells, myeloid-derived suppressor cells, and macrophages. This review presents evidence concerning the impact of arginine metabolism and arginase activity in the prostate cancer microenvironment, highlighting the recent advances in immunotherapy, which might be relevant for prostate cancer. Even though further research is required, arginine deprivation may represent a novel antimetabolite strategy for the treatment of arginine-dependent prostate cancer.

## 1. Introduction

Prostate cancer (PCa) is the second most common cancer in men worldwide [1] and the fourth most incident malignant neoplasia in Europe [2]. As in other neoplasias, prostate cancer is a slow-growing tumor that develops over decades, biologically heterogeneous, and with variable clinical manifestations [3,4]. The limited capacity of aggressiveness stratification using current biomarkers along with incomplete responsiveness to therapeutic options further embodies PCa complexity and advises the quest for additional mechanistic insights and new therapeutic targets.

PCa arises in differentiated epithelial cells and/or progenitor cells as a result of the complex interplay between genes, cellular microenvironment, and host environment [5]. Indeed, the aggressive and metastatic phenotypes of PCa are supported by other mechanisms beyond somatic genetic alterations [6]. The molecular and cellular factors intervening in tumor–stroma interaction may yield improved management of aggressive prostate cancer [7] and add further evidence concerning diet and supplements [8].

Over the last decade, attention has been drawn to arginine metabolism regarding its role in essential processes such as inflammation, cell activation, and cell growth [9,10], which are common features in tumorigenesis. Arginine metabolism is related to the activity of nitric oxide synthase (NOS) isoenzymes and nitric oxide (NO) synthesis, arginase activity, and proline and polyamines synthesis and is implicated in the regulation of transcription of specific genes that modulate free arginine availability [11,12]. Noteworthy, besides tumor cells, arginine metabolism influences many important cellular components at the tumor microenvironment, including macrophages and T lymphocytes, contributing towards suppressed immunosurveillance [9,13]. These findings support arginine and arginases as potentially targetable therapeutic options in oncology.

## 2. Overview of Arginine Metabolism

**Figure 1:**
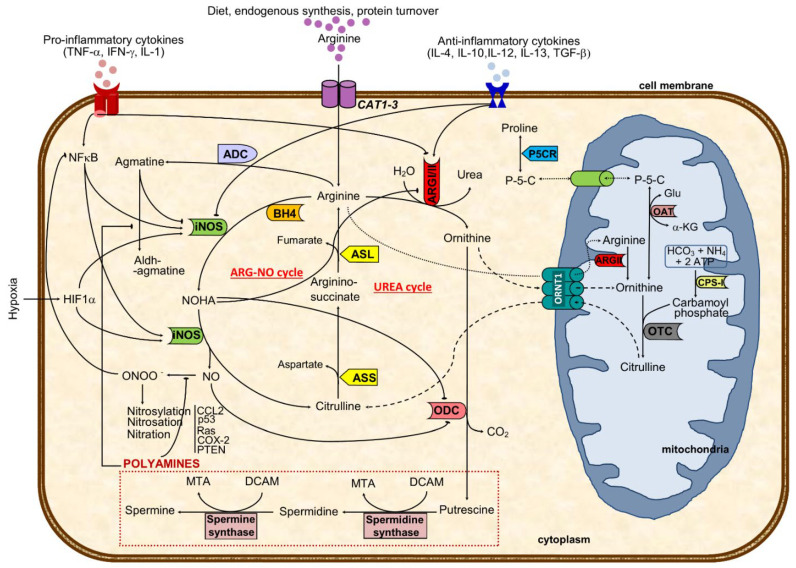
Major cellular arginine metabolic routes and regulatory mechanisms. The aminoacid arginine is transported to the cell by CAT transporters or synthesized from citrulline. Intracellular arginine can either be metabolized by arginase I or II (ARGI or ARGII) to ornithine and urea (excreted out of the cell and of the body) or to NO and citrulline through iNOS catalysis, depending on iNOS/ARG ratio. ARGI expression and activity are regulated positively by anti-inflammatory and negatively via pro-inflammatory cytokines and NOHA, an intermediate in NO formation. In turn, iNOS activity is negatively regulated by agmatine, a decarboxylated product of arginine, and by anti-inflammatory cytokines, besides the positive pro-inflammatory and hypoxic stimulus. Preferential production of ornithine through ARG catalysis can either result in activation of the polyamines pathway (putrescine, spermidine, and spermine) or in ornithine shuttling to the mitochondria (where it might follow the pathway towards proline formation or be transformed in citrulline and shuttled back to the cytoplasm). Arginine antiported to the mitochondria will increase the ornithine pool after ARGII catalysis. When iNOS metabolizes arginine, the resulting products are citrulline and nitric oxide using BH4 as a co-factor. While NO might be transformed to peroxynitrite that may nytrosylate/nitrosate/nitrate key proteins to become pro-tumoral, citrulline formation will drive the ARG-NO recycling. Citrulline from UREA or ARG-NO cycles is converted to arginine-succinate (by ASS), which will result in arginine and fumarate (catalyzed by ASL). This last step represents a link with the Krebs cycle. Solid black lines with arrows indicate a positive effect, whereas solid lines with blunted ends specify an inhibitory effect. Dashed line indicates ornithine-citrulline antiport through mitochondrial membrane, whereas dotted line represents transportation of arginine into the mitochondria or transportation of P-5-C between cytoplasm and mitochondria. ASL, argininosuccinate lyase; ASS, argininosuccinate synthase; CPS-I, carbamoyl phosphate synthetase I; OAT, ornithine aminotransferase; ARG, arginase (type I and type II); ARG-NO cycle, arginine-nitric oxide cycle where iNOS is primarily involved in arginine metabolism; ADC, arginine decarboxylase; ODC, ornithine decarboxylase; OTC, ornithine transcarbamylase; iNOS, inducible nitric oxide synthase; L-HydroxyArg, L-hydroxyarginine; NO, nitric oxide; P-5-C, pyrroline-5-carboxylate; P5CR, pyrroline-5-carboxylate reductase; CAT1-3, cationic amino acid transporters (CAT1 is found in epithelial cells and CAT2 in macrophages); CCL2, Chemokine (C-C motif) ligand 2 or monocyte-chemoattractant protein 1; HIF1α, hypoxia-inducible factor 1 α; NFκB, nuclear factor κB; ORNT1, mitochondrial ornithine: citrulline antiporter; BH_4_, tetrahydrobiopterin; DCAM, decarboxylated 5-adenonosylmethionine; Glu, glutamate; MTA, methylthioadenosine; NOHA, N-hydroxy-L-*arginine*; α-KG, α-ketoglutamate; p53, tumor protein 53; Ras, Ras oncogene; COX-2, cyclooxygenase 2; PTEN, phosphatase and tensin homolog; UREA cycle, urea cycle where ARGI is primarily involved in arginine metabolism.

Arginine, the substrate of arginase, is a nonessential cationic amino acid obtained from diet, endogenous synthesis, and protein turnover and is essential for protein synthesis and a precursor of several molecules such as urea, nitric oxide, polyamines, proline, and agmatine, among others [14] (Figure 1).

Interestingly, while minor arginine disturbances might prompt cellular and organ dysfunctions, its metabolism is highly intricate and tightly regulated. It is synthesized in the cytoplasm from citrulline through consecutive reactions catalyzed by argininosuccinate synthase (ASS) and argininosuccinate lyase (ASL) or transported to the cell by cationic amino acid transporters (CATs 1-3) (Figure 1). The combination of CATs compartmentalization with the different isoforms of nitric oxide synthases has been recently reviewed [15].

Then, in the urea cycle (mainly at the liver), arginase converts arginine into urea and ornithine [16,17]. Hereafter, ornithine can either be recycled again into arginine through the urea cycle or converted to polyamines (by ornithine decarboxylase, ODC) or shuttled to the mitochondria where it is used for proline synthesis (via ornithine aminotransferase) (Figure 1) [18,19]. The rate-limiting enzyme ODC, the first in the biosynthesis of the polyamine that converts ornithine into putrescine, has been shown to facilitate tumorigenesis and invasiveness, being highly expressed in tumors [18,20]. In mitochondria, ornithine is catalyzed by ornithine aminotransferase to pyrroline–5-carboxylate, which is shuttled back to cytosol and reduced to proline (Figure 1). The pyrroline–5-carboxylate-to–proline conversion can transfer the redox potential across the mitochondrial membrane and is involved in several processes, such as protein phosphorylation, platelet activation, cell signaling, hypoxia, cell growth control, and collagen production, which are all relevant in cancer [19,21].

The enzymes ASS, arginase type I (ARGI) and type II (ARGII), inducible nitric oxide synthase (iNOS), arginine decarboxylase, and the CAT activities have all been shown to impact arginine synthesis and catabolism [17,22,23] significantly. Arginine is hydrolyzed by arginase, which has two different isoforms encoded by distinct genes: ARGI, with cytosolic localization and highly expressed in the liver, thought to be mainly responsible for synthesizing urea; ARGII, a mitochondrial or cytoplasmic enzyme with wide expression (including in the prostate), and primarily linked to polyamines, ornithine, proline, glutamate, and NO generation [22,24].

Arginases are encoded by each of their own genes and present almost 100% homology in critical functional areas, with identical three-dimensional crystal structures [25,26,27]. The different properties of the two ARGI/II isoforms are summarized in Table 1 [25,26,28,29,30]. The promoter region of *ARG* genes is stimulated by T helper (Th)-2 cytokines (interleukin-(IL)-4, IL-4; IL-10; IL-12; IL-13) downstream signaling, and by nuclear factors responsive to hypoxia, hypoxia-inducible factor 1 alpha, activator protein, and CCAAT/enhancer-binding protein [24,31,32,33] [23,34]. Arginase activity has been shown to be further regulated by other factors, including androgens, lipopolysaccharide, cyclic AMP, and glucocorticoids [23], as demonstrated in macrophages, T cells, and tumor cells [26,28,29,35,36].

Compartmentalization of enzymes has implications in arginine metabolism. Co-localization of ARGI and ODC in the cytosol guides ornithine usage as a substrate for polyamine synthesis. Conversely, the mitochondrial co-localization of ARGII and OAT, directs ornithine as a preferential substrate for proline and glutamate synthesis (Figure 1) [23,37].

In the prostate, as in the small intestine and lactating mammary gland, ARGII co-localizes with both ODC and ornithine aminotransferase and is related with polyamine and proline synthesis, respectively [38]. When arginase is concomitantly activated with inducible NOS (iNOS), competition occurs, preventing iNOS activity and high NO production [26]. In fact, arginase can downregulate NOS activity by depleting the substrate arginine, being a rate-limiting factor in NO generation [29,33,39,40]. Moreover, arginine may also be catalyzed by arginine decarboxylase to agmatine, a weak competitive inhibitor of NOS that suppresses NO synthesis [18,41,42] (Figure 1). Noteworthy, agmatine may shift metabolism from arginine/iNOS/NO to /arginine/arginase/ODC/polyamine axis, assuming relevance in inflammation and tumorigenesis [42,43], or functioning as a mechanism to regulate anti-proliferative NO and pro-proliferative polyamine effects during inflammation by conversion to its aldehyde [18,41] (Figure 1).

**Figure 2:**
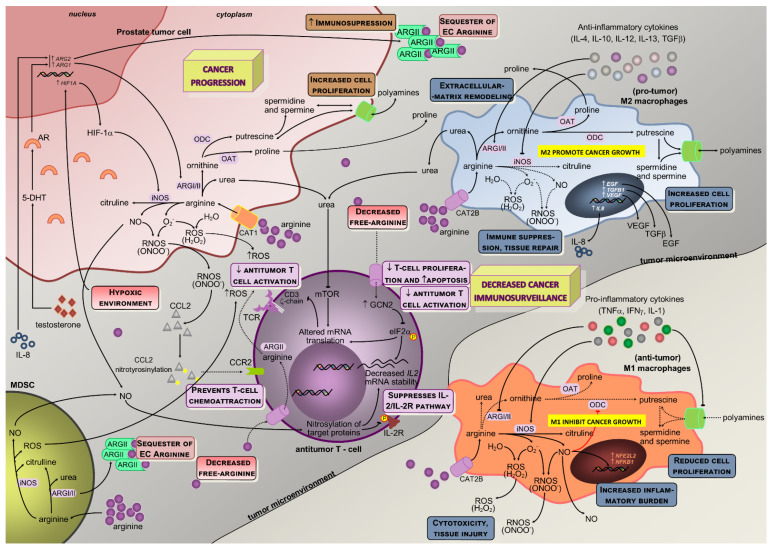
Schematic representation of arginine metabolism and the interplay between cellular players in the prostate tumor microenvironment. The activities of ARGs and iNOS are illustrated, together with arginine-activated downstream pathways in cellular components of the tumor microenvironment (androgen-responsive prostate cancer cell, macrophages, antitumor T-cell, MSCs). The most relevant pathophysiological implications of arginine metabolism are reduced cancer immunosurveillance and a stimulatory action in prostate malignant cells towards cancer progression. Solid black lines with arrows indicate the main enzymatic activity or movement of molecules, whereas dashed lines indicate alternative metabolic pathways or suppression of the movement of molecules. Solid blue lines with arrows designate a stimulatory effect in enzyme activity, while solid red lines with blunt ends specify inhibition of enzyme activity or transporter activity. While pro-tumoral M2 macrophages present increased activity of ARGs with subsequent proline and polyamines production that result in collagen deposition and higher cell proliferation, the antitumoral M1 macrophages (commonly found out of the tumor microenvironment) have an overactive iNOS pathway with resulting pro-inflammatory stimulus (*NRF2L2* and *NFKB1* overexpression), reduced cell growth and production of reactive oxygen (hydrogen peroxide) and nitrogen species (peroxynitrites) that ultimately induce cytotoxicity and tissue injury. The iNOS and ARGs enzymes are tightly regulated by cytokine and metabolic circuits, although these enzymes also directly activate biochemical circuits that negatively regulate each other. As a resulting effect of arginine metabolism and capture in M2 macrophages, several pro-tumoral growth factors are produced and secreted to the tumor microenvironment (VEGF, EGF, TGFβ), while reducing the extracellular pool of free arginine that will contribute to their immune suppression and tissue repair phenotype. By producing IL-4, IL-10, and TGF-β1 anti-inflammatory cytokines, tumor cells also contribute not only to macrophages differentiation towards M2 but also to the regulation of arginine metabolism in macrophages (activating ARGs and downregulating iNOS). Together with tumor cells and MDSCs, macrophages also contribute to increased levels of urea as a result of higher ARGs activity in these cells, which will impair mTOR signaling and T-cell receptor CD3 ζ-chain subunit mRNA translation, thus resulting in hindered antitumor T-cell activation. The hypoxic tumoral microenvironment increases *HIF1A* expression and protein production by tumor cells, which signals downstream to increase arginine-iNOS pathway activation that results in increased production of nitric oxide, and reactive nitrogen and oxygen species. While NO can impact antitumor T-cell activation through nitrosylation of target proteins and suppression of the IL-2/IL-2R pathway, peroxynitrites may hamper CCL2 binding and prevent T-cell chemoattraction towards tumors, and reactive oxygen species can influence negatively antitumor T-cell activation. Moreover, the particularity of prostate tumor cells’ dependence on androgens implies its influence in *ARG1* and *ARG2* expression, which will potentiate the urea cycle towards polyamines and proline production with resulting increases in cell proliferation and collagen synthesis. In addition, the overexpression of *ARG1* and ARG2 will lead to ARGs exportation out of the cell, where they might sequester extracellular arginine, further increasing immunosuppression. MDSCs also metabolize arginine either through the ARG-NO cycle or the UREA cycle (Figure 1). MDSCs in the tumor microenvironment might secrete NO, reactive oxygen species, and ARGs receptors out of the cell, which will contribute to the suppression of IL-2/IL-2R pathway of T-cells, decreased activation of antitumor T-cells and sequestering of extracellular arginine, respectively, ultimately leading to reduced cancer immunosurveillance. 5-DHT, 5alpha-dihydrotestosterone; AR, androgen receptor; *ARG1*, gene coding for the arginase type I; *ARG2*, gene coding for the arginase type II; CAT, cationic amino acid transporter; CCL2, chemokine-CC motif-ligand 2; CCR2, chemokine-CC motif-receptor 2; *EGF*, epidermal growth factor; eIF2α, eukaryotic initiation factor 2α; GCN2, general control of nutrition; *HIF-1α*, hypoxia inducible factor 1 alpha; *HIF1A*, gene coding for the hypoxia inducible factor 1 alpha; IFNγ, interferon γ; IL-1, interleukine 1; IL-10, interleukine 10; IL-12, interleukine 12; IL-13, interleukine 13; *IL2*, gene coding for the interleukine 2; IL-2, interleukine 2; IL-2R, interleukine 2 receptor; IL-4, interleukine 4; IL-8, interleukine 8; iNOS, inducible nitric oxide synthase; MDSC, myeloid-derived suppressor cells; mTOR, mammalian target of rapamycin; *NFE2L2*, gene coding for the nuclear factor erythroid 2-like 2 (Nrf2); *NFKB1*, gene coding for the nuclear factor kappa-b subunit 1; NO, nitric oxide; OAT, ornithine aminotransferase; ARG, arginase (type I and type II); ODC, ornithine decarboxylase; RNOS, reactive nitrogen species; ROS, reactive oxygen species; TCR CD3 ζ-chain, CD3 ζ-chain in T cell receptor; TCR, T-cell receptor; TGFβ, transforming growth factor beta; TNFα, tumoral necrosis factor α; *VEGF*, vascular endothelial growth factor.

Nitric oxide is a simple gas molecule with many roles in cancer-related mechanisms [40,44,45]. It has an early role in cell signal transduction pathways with potentially relevant outcomes and is one of the most relevant arginine metabolism end-products [39,46]. Previous studies identified arginine as a precursor for mammalian nitrite/nitrate synthesis, resulting in the arginine/nitrate-nitrite pathway in macrophages and endothelial cells, which targets the cell function regulator guanylate cyclase [23,36,47]. Tumorigenesis is associated with impaired and/or overexpressed NO, even though NO-mediated apoptosis through caspases activation is frequently observed [44,48]. Notably, consequences of decreased availability of arginine go beyond decreased NO production, since it is also associated with higher NOS-mediated superoxide production [24,49] (Figure 2).

## 3. Arginine Metabolism and Cancer: Regulation of Tumor Microenvironment

High arginase activity has been described in different malignancies, including of the skin, colon, breast, hematologic, and prostate, mainly due to their need to produce polyamines and respond to rapid proliferation [50,51,52,53,54,55]. In agreement, tumor cell lines (some resistant to ODC inhibitors) holding arginase activity resulted in lower production of polyamines, reduced cell proliferation, and induction of apoptosis after inhibition of ARG activity [56,57].

Besides the mechanism of substrate deprivation for cancer growth, in which arginases and arginine availability have a nearly direct effect in cancer cells, current perspectives elicited an extended explanation focused on the interplay of arginases metabolism and arginine among components of the tumor microenvironment as mediators of immunosurveillance. Notably, the efficacy of chimeric antigen receptor T cells (CAR-T) in hematological and solid malignancies has been shown to be compromised by depletion of arginine due to the low expression of arginine resynthesis enzymes, ASS, and ornithine transcarbamylase; therefore, the re-engineering of T cells may correct the mentioned defect and induce the expression of functional ASS or ornithine transcarbamylase enzymes, increasing CAR-T cell proliferation without compromising function [58].

Arginine depletion exerted target transcriptional and posttranscriptional regulation of ASS and ASL activities and regulation of other genes such as those coding for iNOS, CAT-1 or T-cell receptor (TCR) zeta-chain, thereby modulating relevant cellular mechanisms (Figure 2): (i) there is a diminished efficiency in iNOS mRNA translation and protein stability triggered by lower extracellular arginine and arginases overactivity due to the downregulation of eukaryotic initiation factor 2α (eIF2α) [18,23,42]; (ii) the lower TCR zeta-chain mRNA stability and expression affects lymphocytes proliferative capability [29,59]; (iii) both transcriptional and translational efficiency of CAT-1 mRNA are increased [24,29], conferring advantage for capturing extracellular arginine to tumor cells. During tissue repair, ornithine conversion into polyamines (putrescine, spermidine, and spermine) might be upregulated, inducing cell growth and proliferation, as well as different downstream signal transduction pathways (e.g., inhibition of pro-inflammatory cytokine secretion) [18]. In this setting, arginine deprivation induces compensatory polyamine transportation, thus modulating polyamine synthesis and NO metabolism [18,23].

During tumor progression, macrophage polarization switches from classically activated M1, which promotes tumor initiation and adaptive immunity activation towards an alternatively activated (M2) polarized state, favoring tumor progression and spread [60]. In M1, inflammatory mediators regulate ASS expression together with iNOS, ASL, and CAT-2 activities, thus providing increased arginine synthesis and uptake for NO production. This regulation of arginine and NO by inflammatory mediators is also found in other cell types (e.g., epithelial cells and vascular smooth muscle) and establishes the arginine-NO pathway that supports NO generation (Figure 1) [19,24]. M1 macrophages may contribute to the eradication of cancer cells once they generate NO, pro-inflammatory cytokines, and chemokines, or may act as antigen-presenting cells to activate CD8+ cytotoxic T-cells [61]. Conversely, M2 macrophages promote tumorigenesis through anti-inflammatory cytokines and chemokines (IL-4, IL-13, and transforming growth factor β, TGF-β pathways) [61] (Figure 2). Moreover, this M2 phenotype is also related to decreased production of chemoattractants that stimulate migration of inflammatory cells toward tumors, further suppressing the inflammatory burden and promoting tumorigenesis [61]. These M2 macrophages promote debris clearance, angiogenesis, tissue remodeling and repair, sustaining wound healing, tumor cell survival, and disease progression [62,63,64]. Interestingly, while M2 macrophages present modified arginine metabolism toward proline and polyamine synthesis, M1 macrophages have increased citrulline and NO levels, with higher iNOS expression [9,55,65] (Figure 2). Findings from studies on tumor-associated macrophages (TAMs) with an M2-like phenotype indicate that iNOS/ARG balance within macrophages is relevant for tumor progression [9,55]. In aging prostates, the TAM was restricted to areas of premalignant and malignant lesions and is therefore putatively involved in cancer progression [66].

In macrophages, both ARGI and ARGII and NOS expression and activity were identified [31,33]. ARGI is markedly induced in human mononuclear cells after tissue injury, associating with decreases in arginine and NO availability [33,39]. ARGI is inducible, while ARGII is constitutively expressed in macrophages and tightly regulated by Th1 and Th2 cytokines that modulate the production of ornithine and subsequent products [32,33]. Indeed, pro-inflammatory cytokines (tumor necrosis factor alpha, TNF-α; interleukin 1, IL-1; interferon gamma, IFN-γ) induce iNOS and suppress arginase activity, whereas anti-inflammatory cytokines (IL-4, IL-12, IL-13, and IL-10) reduce iNOS and increase arginase activity [18,21,32] (Figure 2). As in epithelial cells, also in macrophages, the synthesis of NO is dependent on arginase expression, which is well correlated with arginase activity [21,39,67]. Further, citrulline and NO are produced by tumor cells by catabolizing arginine [68]. Increased arginase activity induces a phenotype in macrophages that favors tumor cell growth, through the provision of ornithine for polyamine, proline, and collagen synthesis and suppression of tumor cytotoxicity by reducing NO production [19,23,24,26,33].

The key inflammation-related transcription factors nuclear factor erythroid 2-like 2 and nuclear factor kappa-B can be modulated by NO in malignant cells promoting cell survival and tumor progression [69]. Even though tumor cell-induced iNOS is involved in those processes through NO production, its activation in macrophages further increases the inflammatory burden [36,65]. Additionally, arginase induction in macrophages decreases NO production by conversion of arginine in ornithine [22,36,39,46,48]. In this process, ARGII and ODC are initially induced and may lead to increased polyamine production [33]; and then followed by iNOS downregulation, further relocating arginine to ornithine synthesis that can be used for polyamines and proline synthesis, essential for cell proliferation and tissue remodeling [18,21,33,39,70]. Notably, hypoxia seems to influence arginine metabolism in TAMs, resulting in suppression of adaptive immunity and reduced tumor immunosurveillance [23,48]. Alternatively, M1 macrophages, where iNOS expression is increased, are more likely to induce DNA damage, lipid peroxidation, nitration of tyrosine residues, and oxidation of thiols [18,33] (Figure 2).

The lower availability of arginine in the microenvironment that results from upregulated arginine metabolism in tumor cells, in M2 macrophages, and in myeloid-derived suppressor cells (MDSCs), exerts a regulatory effect in lymphocyte activation through downregulation of the TCR CD3ζ chain, central for signaling and responsiveness in activated T cells [71]. It was shown that arginase upregulation, together with CAT-2 transporter under expression in tumor-surrounding cells, reduces arginine availability and downregulates CD3ζ T cell receptor expression [29,33,71], with an inhibitory impact on antitumor T cell activity. This mechanism might be used by anti-inflammatory macrophages, M2-like, to modulate T-cell function in the tumor microenvironment [59]. Taken together, these results suggest that decreased arginine availability and increased arginine metabolism in cells adjacent to lymphocytes may have a suppressive role in the cell-mediated immune response to cancer.

The complex crosstalk within tumor microenvironment cellular players is mediated by several tumor-derived cytokines (e.g., vascular endothelial growth factor, VEGF; interleukin 1 beta, IL-1β; granulocyte/macrophage colony-stimulating factor, GM-CSF), which also have a role in immunomodulation [72]. The signaling pathways activated by tumor-derived factors allow immature myeloid cells such as MDSCs to inhibit T cell function through induction of apoptosis, inhibition of cell proliferation, and expressing regulatory phenotypes [72]. Accordingly, M2-like macrophages are characterized by secreting high levels of immunosuppressive cytokines (IL-10 e TGF-β) that inhibit immunological-mediated antitumoral response [64]. This phenotype delays the maturation of tumor-associated dendritic cells contributing to an increased pool of immature myeloid cells, which impair antitumoral T cell activity [61,62,64,65,73]; therefore, as shown, the setting of a fitting tumor-modulated microenvironment can shift the immune response in a favorable way to facilitate tumor growth.

Morphologically and functionally heterogeneous MDSC secrete high amounts of IL-10 that preferentially differentiate macrophages to an M2 phenotype [61,65,74]. These cells are found in the patient’s tumor microenvironment and lymphoid organs due to their ability to suppress innate and adaptive antitumoral immunity (inhibit CD4^+^ and CD8^+^ T cells, and block dendritic cell maturation) [61,62]. Both iNOS and ARGI can be expressed at the same time in MDSCs, under different stimuli, generating a suppressive mechanism where superoxide levels are increased through an iNOS-mediated pathway [62] (Figure 2). Superoxide is required to modulate T cell suppression [62]. The presence of MDSC positive for ARGI is associated with compromised antitumor cytotoxic T lymphocytes [61,65]. Briefly, these cells express both ARGI and iNOS that modulate T cells response through depletion of arginine, leading to peroxynitrite production, inhibition of CD3ζ expression in T cells, and induction of apoptosis [62,65].

## 4. Arginine Metabolism in Prostate Cancer Tumors

Increased arginase expression and activity have been reported in many tumors, such as head and neck, kidney, breast, hepatocellular, and prostate [28,54,75,76,77]. In fact, in prostatic diseases, arginine metabolism and arginase activity arise as natural subjects of interest since arginase expression and polyamine synthesis were found to be elevated in prostate cancer and associated with differentiation [13,28,50,78,79,80]. Besides tissue, few and low-powered studies observed distinct arginase activity in prostate cancer and non-prostate cancer serum samples [81,82]. Immunohistochemical studies in human samples showed increased ARGII expression in localized and well-differentiated androgen-dependent tumors, along with benign prostatic hyperplasia, prostate intraepithelial neoplasia, and normal tissue, compared with more aggressive tumors [24,28,50,80]. The pathophysiological foundations for these findings may rely on the elevated demand for ornithine by neoplastic cells to produce polyamines for tumor progression [29,83]. This was further confirmed in vitro by adding recombinant human arginase to the culture medium of PCa cell lines, which resulted in significant arginine deprivation and consequent cytotoxicity through an effect in ornithine carbamoyl transferase expression and inhibition of the mammalian target of rapamicin (mTOR) [84].

Arginine starvation in prostate and breast cancer cells induced mitochondrial dysfunction, depletion of mitochondrial metabolites, alteration of mitochondrial morphology, and generation of mitochondrial reactive oxygen species [85]. Further, DNA damage and excessive autophagy are accompanied by the silencing of nuclear-encoded mitochondrial genes, including oxidative phosphorylation genes and nucleotide synthesis genes [85]. TEAD-4, a family of transcription factors, located in mitochondria, is involved in the regulation of mitochondria-encoded genes involved in oxidative phosphorylation activities. Moreover, arginine is an epigenetic regulator targeting TEAD4 to modulate oxidative phosphorylation in PCa cells [86].

However, arginine is still not a suitable substrate compared with citrulline due to limits on systemic availability [87] Interestingly, the addition of exogenous L-arginine to the culture medium with T cell increased intracellular levels of free L-arginine and induced a metabolic switch from glycolysis to oxidative phosphorylation [88]. Nevertheless, inhibition of arginases in human T cells or deletion of ARGII in mouse T cells did not affect cell proliferation [89].

A radiotherapy is a therapeutic option for patients with localized prostate cancer. Using a murine model, it was shown that irradiated prostate tumors and TAMs had increased expression of Arg1 and Nos2, and the crosstalk between malignant cells with TAMs conferred increased aggressiveness to the tumor [90]. So far, few studies have demonstrated the expression and/or activity of ARGI and ARGII in malignant prostatic cells, according to their dependence on androgens. Both isoforms were expressed in androgen-dependent and androgen-independent prostate cancer cells, although in androgen-dependent presented higher ARGII activity compared to androgen-independent prostate cancer cell lines [28,50]. It was shown in androgen-dependent cells that both ARGI and ARGII expression are responsible for the activation of immunosuppressive pathways and proliferative stimulus in an androgen receptor-dependent manner [50].

Cumulatively, androgen deprivation therapy resulted in lower ARGII expression in patient’s non-malignant and malignant prostatic epithelial cells [50]. Thus, ARGII seems to promote prostate cancer cell proliferation and induces an immunosuppressive environment in earlier hormone-sensitive stages of prostate cancer [50]. 

## 5. Modulation of Arginine Metabolism in Oncology: From Basic to Clinical Research

Modulation of arginine availability and arginase activity by targeting the enzymes in its metabolic pathway may have a role in cancer therapeutics. The auxotrophic affinity of cancer cells for specific amino acids has been the rationale for therapeutic deprivation regimens, where arginine fits, once it is required by proliferating cells, despite nonessential to normal cells. Downregulation of the rate-limiting arginine-producer enzyme ASS in tumor cells, which is common in most cancers [83], correlates with the dependence on extracellular arginine due to the inability to produce endogenous arginine for growth [56]. Thus, arginine-depleting enzymes involved in arginine catabolism out of the cell (arginase and arginine deiminase, androgen-independent, a microbial enzyme that converts arginine to citrulline and ammonia) may have an antitumor effect, with tumoral ASS deficiency serving as a prognostic biomarker and predictor of sensitivity to arginine deprivation therapy [57].

A pegylated form of androgen-independent (ADI-PEG20) has been tested in melanoma and hepatocellular carcinoma cell lines [91], with encouraging results. Interestingly, ADI-PEG20 has shown promising in vitro and in vivo results on prostate cancer specimens without ASS activity [92], which is a common feature of prostate tumors [83].

Citrullination is a deimination of protein-embedded arginine, which is converted to the non-coded amino acid citrulline [93]. This process is catalyzed by a family of enzymes called peptidyl arginine deiminases. Citrullination is involved in disease pathogenesis with the involvement of the following mechanisms epigenetic, pluripotency, immunity, and transcriptional regulation. Indeed, peptidyl arginine deiminase 2-mediated arginine citrullination might have an implication on transcriptional regulation in cancer [94]. Furthermore, citrullination of histones in neutrophils facilitates neutrophil extracellular trap formation or NETosis [95]. In addition, interleukin-8 (IL-8) induces neutrophil extracellular traps in granulocytic MDSCs in the same way that it induces them in neutrophils [96]. Notably, IL-8 that is upregulated through androgen stimulation also contributes to ARGII expression, even in the absence of androgens [50] (Figure 2).

**Figure 3:**
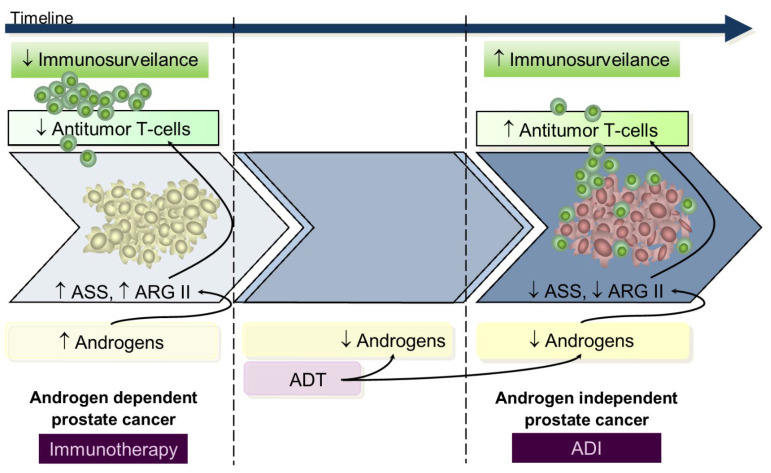
Arginine metabolism according to prostate cancer hormonal status informs plausible therapies. During early AD PCa development, androgens are available and contribute to increased immunosuppression and proliferative stimulus through ARGII mediation. In this setting, increased ASS activity confers resistance to ADI administration, and the most benefit is likely to derive from immunotherapy. Conversely, emergence of the androgen-independent phase of disease, after acquired resistance to ADT therapy, is associated with tumor cell survival in very low androgen levels with subsequent low ASS and ARGII expression and normal immune activity (conferring higher response rate to ADI treatment). ADT, androgen deprivation therapy; ADI, arginine deiminase; ARGII, arginase II; ASS, argininosuccinate synthase.

Data from in vitro and in vivo studies using either in androgen-dependent or androgen-independent prostate cancer cell lines suggests that during the androgen-dependent phase, with higher ASS and ARGII expression, tumors were resistant to androgen-independent, whereas in the androgen-independent stage, presenting decreased ASS and ARGII expression, there was a response to androgen-independent treatment [50,92]. From this perspective, we hypothesize that during the early androgen-dependent PCa development preeminent mechanisms contribute to immunosuppression rendering immunotherapy a key role; on the contrary, advanced androgen-independent tumors that survive in low androgen environments have increased T-cells infiltrated [97] and low ASS and ARGII expression [50,92] rendering androgen-independent a potential therapeutic utility since lymphocyte activity is already restored (Figure 3); however, despite these findings from in vitro and animal models [56,98,99,100], and in patients with metastatic cancer [101,102,103], the apparently low potency of ARG and AD androgen-independent immunogenicity advise restrained enthusiasm. Tumor cell resistance mechanisms to androgen-independent have already been uncovered [104]. An updated detailed review of clinical studies using arginine-degrading agents has been published [105].

Immunotherapy has been perceived as a promising approach in treating various cancer types, including prostate tumors [106,107]. As mentioned earlier, MDSCs interfere with the adaptive immune antitumor response by suppressing T cell activation through unbalanced arginine metabolism via iNOS and ARGI. This effect leads to peroxide generation, lack of CD3ζ chain, T cell apoptosis, tumor progression, invasion, and metastasis. Maintenance of arginine baseline levels and decreased arginase expression allow tumor-specific CD8^+^ T cells and cytotoxic M1 macrophages activation, delaying the metastatic disease. The stimulation with an Arg-1- derived peptide (ArgLong2, 38-mer, positions 169–206 in Arg-1) in vitro, strongly impacted responses against Arg-1 in healthy donors and cancer patients, rebalancing the microenvironment [108]. Conversely, deletion of Arg-2 in T cells significantly reduced tumor growth in preclinical cancer models by enhancing CD8+ T cell activation, effector function, and persistence. Noteworthy, specific deletion of Arg2 in CD8+ T cells strongly synergized with PD-1 blockade for the control of tumor growth and animal survival [35]. The arginase isoform expressed by T cells, the mitochondrial Arg2, is an intrinsic regulator of CD8+ T cell activity. The increase in L-arginine levels may induce global metabolic changes, including a shift from glycolysis to oxidative phosphorylation in activated T cells and promote the generation of central memory-like cells endowed with higher survival capacity [109]. In PCa, various studies reported increased expression and activity of iNOS and ARG [50,110], while others assessed the benefit of selective antagonists for ARGI and iNOS in restoring T-cell mediated cytotoxicity [62,65]. It was already shown that the use of NOS and/or ARG inhibitors would upregulate activation of CD8^+^ antitumor lymphocytes, restoring their functionality and survival [62,64,65,111,112]. Strategies involving the administration of ARGI inhibitors to recover M1 immunity might reactivate the tumor-specific Th1 immunity, and regain the cytotoxic activity [65].

## 6. Conclusions

The complexity of arginine metabolism, which involves many and compartmentalized isoenzymes crosstalk, and the intricate regulatory network with distinct players from the tumor microenvironment depict an overall intriguing yet exciting picture. Arginase has a role in cancer pathophysiology, although other enzymes, molecules, and transporters of arginine metabolism should also be considered. Arginine availability and arginase activity have been consistently related with two main pro-tumoral mechanisms: (1) NO, polyamine, and proline synthesis that are associated with cancer progression, (2) activation of immunological effector cells, decreasing tumoral immunosurveillance.

Strategies to modulate arginase activity with impact on M2-to-M1-like macrophage polarization, regulation of effector immune response, and inhibition of evasion might reveal a useful approach for prostate cancer therapy. The growing body of knowledge on arginase structural, functional, and integrated aspects fostered our understanding of the association between arginase and arginine metabolism with prostate cancer. These mechanisms have granted the scientific support to be considered a biological factor with interest for oncology, particularly prostate cancer. Nonetheless, the road ahead includes robust clinical validation studies, both at translational and clinical levels.

## Figures and Tables

**Table 1:** Characteristics of mammalian arginase isoforms.

	ARGI	ARGII
Gene size (exons)	8	8
Noticeable genetic features	C/EBP elements (at −90 and −55 bp)	Probable LXR-response element
Chromosome region	6q23	14q24.1-24.3
Amino acids residues	322, 323	~333
Subunit structure	Trimer	Trimer
Main subcellular location	Cytosol	Mitochondria **
Tissue specificity *	Liver, RBC, submaxillary gland	Kidney, small intestine, brain, prostate, lactating mammary gland
Subunit molecular mass (KDa)	35	40
pI	9.7–9.9	9.4–10.0
[Mn^2+^] requirement–cofactor (nmol/L)	20	2
Km (arginine)	5.9 nM	7 nM
Inhibitors	NOHA, valine	NOHA

ARGI, Arginase I; ARGII, Arginase II; C/EBP, CCAAT-enhancer-binding proteins; LXR, liver X receptor family; NOHA, N-hydroxy-L-arginine; RBC, red blood cells. * Although both arginase isoforms might be found in almost any tissue, this specificity reports to the tissues where higher amounts were found. ** Besides mitochondria, ARGII might be found in cytoplasm or in the extracellular space.
